# Artificial intelligence in modern clinical practice (Review)

**DOI:** 10.3892/mi.2025.289

**Published:** 2025-12-09

**Authors:** Krupa Sara Thomas, Sudeep Edpuganti, Divina Mariya Puthooran, Angela Thomas, Angel Joy, Shifna Latheef

**Affiliations:** Department of Medicine, Faculty of Medicine, Tbilisi State Medical University, Tbilisi 0177, Georgia

**Keywords:** artificial intelligence, machine learning, deep learning, clinical decision support, medical imaging, digital pathology, robotic surgery, cardiology, precision medicine, healthcare ethics

## Abstract

Since its inception as rule-based programs, artificial intelligence (AI) has developed into machine learning and deep learning systems that utilize the enormous volumes of clinical data currently accessible. The aim of the present review was to discuss the role of AI in modern clinical practice and to highlight the opportunities and challenges that lie ahead by combining the results of recent research. AI tools provide physicians with decision support and prediction models, directs robotic procedures and surgical planning, supports radiologists, pathologists, dermatologists and ophthalmologists with image analysis, and aids in the delivery of more individualized care in cardiology and precision medicine. These developments are boosting precision, optimizing daily tasks and providing patients with more individualized treatment. In practice, this could include imaging systems that prioritize patients who are most at risk or prediction technologies that help physicians allocate resources and reduce unnecessary workload. However, there are still critical obstacles to overcome. The biases of the training data may be reflected in the algorithms, which could exacerbate already-existing disparities. Since many models operate as ‘black boxes’, it can be challenging to understand their logic, which raises questions about accountability, ethics and trust. Clinical standards and regulations are still lagging behind technology, and incorporating AI into busy healthcare systems can be difficult and costly. Achieving its promise will require careful implementation, rigorous validation and sustained collaboration among clinicians, data scientists, engineers, ethicists and policymakers for safe adoption in clinical practice.

## 1. Introduction

Artificial intelligence (AI) in healthcare refers to computer systems that perform tasks typically requiring human intelligence. Deep learning (DL) describes complex patterns using multi-layer neural networks. Machine learning (ML) is a subfield that involves algorithms that learn and get better from data (1). Natural language processing (NLP) is another key field that allows computers to comprehend and produce human language. These AI techniques power diverse applications, from automated image interpretation to clinical decision support and even robotic-assisted surgery (2) that are transforming modern medicine.

AI in medicine has developed over a number of years. Early rule-based medical expert systems included INTERNIST-1 (1971) and MYCIN (1970s), which were developed to support diagnostic decision-making (2). Over time, AI moved from these knowledge-based programs to statistical and neural models. Recent years have seen an explosion of data [from electronic health records, Internet of Things (IoT) devices, genomic databases, etc.] and advances in computing power. This highly structured environment has led to a rise in the use of AI. DL and ML are increasingly being utilized to examine large clinical datasets for tailored treatment and diagnosis. For instance, Aamir *et al* (3) pointed out that big data and the IoT have greatly increased the importance of AI by generating large amounts of health data that necessitate machine learning analytics.

Currently, AI is widely recognized as a transformative tool in medicine. AI can ‘fundamentally transform the practice of medicine and the delivery of healthcare’, according to Bajwa *et al* (4). AI has the potential to improve patient outcomes by increasing medical efficiency and diagnostic precision (2). A recent multicenter study found that a DL ECG model (EchoNext) outperformed cardiologists at detecting structural heart disease from 12-lead ECGs (model accuracy 77.3% vs. cardiologists' mean accuracy 64.0%; sensitivity 72.6% vs. 61.1%; specificity 80.7% vs. 66.1%; reader study: 150 ECGs read by 13 cardiologists), demonstrating that AI-driven tools can surpass human experts in certain ECG diagnostic tasks (5). Similarly, in domains such as radiology, dermatology and pathology, AI algorithms used for medical imaging have performed on par with or better than humans (2). By reducing routine errors and workload, AI holds promise to advance the healthcare ‘quadruple aim’ of improved outcomes, improved patient experience, lower costs and enhanced provider satisfaction (2,4).

The aim of the present review was to summarize and discuss the role of AI in modern clinical practice and to highlight the opportunities and challenges that lie ahead by combining the results of recent research.

## 2. AI in diagnosis and prognosis

AI is transforming medical imaging by improving workflow efficiency, prognostic forecasts and diagnostic accuracy. DL and neural networks help with the advancements (6). Crucially, AI enhances diagnostic accuracy and supports the talents of radiologists in a team environment, complementing rather than replacing doctors (7).

### Imaging and radiology

By identifying complex patterns in large imaging and clinical datasets, AI enhances radiology diagnosis and risk assessment (8). By supporting research, treatment planning, risk assessment and case identification, it expedites the delivery of healthcare (8). AI facilitates individualized treatment and eventually enhances patient outcomes by identifying subtle visual aspects (9).

Convolutional neural networks (CNNs) are advanced neural networks without manual intervention to capture patterns from visual data. The model recognizes subtle patterns which are optimal for medical image and disease detection (10). CNNs employ neural network architectures with many layers that construct relevant features automatically and carry out feature construction and prediction within the same model (9).

*Lesion detection*. CNN-based technologies, such as Auto Lung Nodule Detection (ALND) detect and categorize lung nodules (11). These technologies achieve high accuracy in differentiating benign from malignant nodules (6,7). When compared to radiologist reports, the program exhibits a low positive predictive value (5.5%), but a high specificity (83.2%) (11). CNNs acquire structured attributes such as boundaries, patterns and configurations straight from the visuals. The multiscale feature records tiny nodules and adjacent tissues providing more sensitivity and minimizing irrelevant data (10). A comparison of the performance of AI vs. that of human experts across different specialties is depicted in Table SI.

*Automation and triage*. Routine image analysis and report generation are automated by AI, including NLP (6). Additionally, it gives priority to urgent cases, allowing radiologists to analyze key results more rapidly (6,7,11).

*Advanced imaging*. AI-based CAD systems integrate multimodal imaging (fMRI, MRI, DTI, PET/CT and MR spectroscopy) to localize tumors more accurately (6,12). AI aids surgical planning and reduces damage to healthy tissue by improving image precision. Brain scans can also provide biomarkers of neurodegenerative disorders (6).

*Additional uses*. By identifying minute lesion characteristics, AI improves breast imaging (6,7,12). AI enables organ segmentation and early detection of pancreatic cancer on abdominal imaging (6). It also improves fracture detection and arthritis monitoring. AI models can identify autoimmune diseases: For example, they can detect systemic lupus erythematosus (including neuropsychiatric systemic lupus erythematosus) early and distinguish it from rheumatoid arthritis and multiple sclerosis using biomarker analysis (achieving ~96.5% accuracy) (6,8,12). Small nodules can be missed when overlapping with ribs or blood vessels (10).

### Pathology and digital pathology

The incorporation of AI into pathology has initiated a paradigm shift, enhancing diagnostic precision and workflow (7). AI tools help in detecting, segmenting and analyzing tumor cells with spectacular diagnostic performance, outperforming experts in narrow tasks (13).

*Prostate cancer*. AI technologies reduce the labor of pathologists by pre-screening histology slides to identify malignancy. High accuracy (ROC-AUC ~0.99) in detecting neoplastic lesions is attained by CNNs trained on annotated slides (14). AI tools, for instance, Paige prostate, authorized by the FDA in 2021, and Galant prostate, developed by IBEX in 2020, yielded an excellent sensitivity of 97.7% and specificity of 99.3% in detecting 1876 prostate biopsies (14).

*Grading and prognosis*. Gleason scoring can be aided by AI, as demonstrated in a challenge involving >10,600 biopsies (14). The emergence of digital pathology with whole slide imaging (WSI) digitalization has laid the foundation for AI and ML in pathology. AI can rapidly generate Gleason scores, tumor size, grade and invasion markers, such as perineural invasion and enhance patient care. Workflow efficiency is further increased when AI is integrated with electronic medical records (15). AI significantly used for oncologic detection substantially aids in tailored therapeutics, precisely estimating reactions to therapy and health results, which is by integration a fusion of histopathological, genetic and medical history (13).

*Advanced prognostic models*. DL identifies computational biomarkers that are invisible to humans from genomic and WSI data [e.g., prostate-specific antigen (PSA) recurrence signals] (14). Multimodal AI that combines pathological and clinical/genomic data is more accurate in predicting outcomes (survival, PSA relapse and metastasis) than conventional techniques (14). The multimodal DL AI models trained on 16,204 slides from 5,654 patients surpassed conventional risk scoring tools, such as National Comprehensive Cancer Network (NCCN) risk categories throughout all clinical endpoints, yielding a high AUC from 9.2 to 14.6% (14). Sophisticated AI techniques, such as Vision Transformers and multiple instance learning (MIL) process small patches to evaluate whole slide images (14). AI aids in triaging patients for innovative therapies, resulting in minimizing expense and limiting adversary actions (15). Pathological investigation is accelerated using tissue microarrays, which allow for the simultaneous review of numerous biopsy sites (16).

*AI architectures in pathology*. CNNs are widely used in detecting and breaking down images into multiple layers, which helps to identify whether it is cancerous or non-cancerous, grading tumors, and predicting prognosis with tumor analysis (13). CNNs have also been used in digital pathology for quality control. For example, a CNN-based DeepFocus system has an average accuracy of 93.2% (±9.6%) in identifying and segmenting out-of-focus regions in whole-slide images (15). Fully convolutional networks are used for pixel-wise cancer detection (e.g., identifying rare head/neck carcinomas or breast cancer, ~71% accuracy) (15).

Recurrent neural networks (RNNs) and long short-term memory networks are used for temporal or sequential data in imaging follow-up (15). Generative adversarial networks (GANs) are used for image synthesis and segmentation (e.g., generating renal glomeruli images, predicting PD-L1 from histology) (15). When documented data are constrained, GANs can improve small databases and increase the precision of segmentation through the generation of realistic artificial images and can produce variants of rare lesion types. However, when data are limited, GANs may collapse and may not be reliable. This can create realistic synthetic medical images, but can lack in real-life cases (17,18). GANs comprise two neural networks, a generator that creates synthetic images and a discriminator that evaluates them, trained adversarially; thus, the generator progressively produces more realistic outputs (18). Handcrafted feature methods (e.g., counting mitotic figures) based on domain knowledge, are used alongside data-driven approaches (15).

### Dermatology

The early detection of skin cancer, notably melanoma, markedly enhances patient outcomes and achieves a 5-year survival rate of 99% if detected early (19). A number of skin lesions appear similar, rendering diagnosis challenging for specialists (20). The advent of dermoscopy increased the diagnostic accuracy (from ~65-80 to ~75-84%) (21); however, AI provides further benefits. AI also aids non-specialists: In a previous study with 2,201 images and 134 dermatological conditions, AI was used for verifying the algorithm; 240 images were randomly selected for human trials including 23 non-medical professionals (22) and it exhibited an accuracy of 47.6 to 87.5% with no compromise in specificity (22,23). In frontline healthcare, referral data-based AI models have successfully detected the three leading differential diagnoses with an accuracy of 93% and specificity of 83% in 26 common skin conditions comprising up to 80% of cases in primary care cases, outperforming nurses and primary care physicians (23). A previous systematic review concluded that transfer learning, which is AI-based, rivals in identifying skin disorders (24).

AI currently aids in skin lesion analysis. CNNs are engineered for distinguishing traits such as pigmentation, structure, size and depth, which are vital for correct skin lesion interpretation (25). It has been demonstrated that AI systems can match or exceed experts: In a previous study, one CNN (trained on 12,378 dermoscopic images) outperformed 136 of 157 dermatologists with a mean specificity of 60% and a mean sensitivity of 87.5% (26). An Advanced neural network repeatedly performed on par with clinical experts, confirming AI reliability (27).

Key AI techniques in dermatology include the following: i) The use of models pretrained on large image libraries (e.g., ImageNet) to improve performance on limited dermatology datasets (24); ii) the types of mechanisms, such as self-attention, hard attention and soft attention, guide AI to prioritize image features, mimicking human-like attention (20); iii) merging two CNNs, such as IBR5 and IBR6, to create hybrid networks has achieved 91.3 and 90.7% accuracy, respectively [10,015 histological images (HAM10000, ISIC2018)] (21).

In dermatopathology, WSI and AI enable the prediction of clinical outcomes. Models forecast sentinel lymph node involvement, recurrence risk and mortality from histology (23,28).

### Ophthalmology

AI-based systems (CNNs; EyeArt, IDx-DR, Google Health, Moorfields-DeepMind, Retina-AI, IBM Watson, EyePACS and mobile AI apps) diagnose eye diseases with high accuracy and improved clinical outcomes (29). In diabetic retinopathy, IDx-DR is the approved tool for AI in screening with a sensitivity of 87.4% and specificity of 89.5% (30), which was tested on 900 patients across 10 primary care settings; it yielded a high accuracy and a 96.1% image ability (31). Improved systems such as EyeArt 2.0, which uses fundus screening for diabetic retinopathy, was used on large datasets of ~850,908 images from 101,710 patients, and yielded a high accuracy with a sensitivity and specificity of 91.3 and 91.1%, respectively, further reducing false positives (30). ML systems interpret optical coherence tomography (OCT) and fundus scan can diagnose and stage age-related macular degeneration (AMD) (29). DeepSeeNet analyzes color images to evaluate AMD stage and can outperform retinal specialists. It is trained on 60,000 images and has been tested on 900 images and has outperformed retinal specialists with an accuracy of 0.671 vs. 0.599, a sensitivity of 0.590 vs. 0.512 and a specificity of 0.930 vs. 0.916(30). AI models combining OCT and visual field data detect glaucomatous optic neuropathy at an earlier stage than clinicians (29).

## 3. Decision support and precision treatment

### Decision support

AI aids physicians in making more rapid and accurate diagnoses by analyzing a large number of clinical images, such as chest X-rays, skin lesion images, pathology slides and cardiac data. An autonomous AI tool known as the IDx-DR system approved by the FDA can detect more than mild diabetic retinopathy directly from retinal images. It exhibited high accuracy, with a sensitivity of 87% and specificity of 90% (4). For instance, Stanford University trained a CNN on >112,120 chest x-rays from the National Institutes of Health (NIH) dataset. The AUC for each pathology was used to assess the performance of the model; for atelectasis, the CNN AUC was 0.862 (95% CI, 0.825-0.895), as opposed to 0.808 (95% CI, 0.777-0.838) displaying competitive performance for radiologists (32). Similarly, the study by Hashim *et al* (33) observed a dataset of 120,950 patients to compare the diagnostic accuracy of the AI and radiologist. Their study concluded that AI was more effective at detecting breast cancer with a sensitivity of 0.85, while the sensitivity for physicians was 0.77. However, both exhibited comparable results in the context of specificity (33). In fact, AI can aid in planning a more rapid and more accurate treatment in prostate cancer by automatically identifying clinical target volume. Additionally, the CNN-based model achieved an AUC of 0.85 in detecting rectal cancer based on endoanal ultrasound (34).

When unsure of symptoms, patients can determine the care they need by allowing them to input what they are experiencing into AI tools, which analyze data based on past medical information and medical guidelines and suggest the urgency of the situation (35). Thus, AI prevents unnecessary hospital visits and is helpful in the emergency department by identifying life-threatening conditions early and alerting physicians for timely intervention (35).

Therapeutic drug monitoring (TDM) is a process by which a physician regularly checks certain medication levels in the blood of a patient, which is of particular importance for drugs with a narrow therapeutic window (36). AI assists in predicting the clearance rate of a drug and the effective or toxic dose of a medication by processing information, such as genetics, past medical conditions, age, weight, kidney or liver function and other medications, thus making TDM more precise (37).

Yet another benefit of AI emerging is its ability to predict how different drugs interact with each other and prevent side-effects, and to anticipate who is more likely to have these side-effects (38). Beyond drug safety, AI can identify patients whose conditions are at high risk of deteriorating before the visible symptoms are found, and can positively affect the prognosis. Previously, a significant reduction was observed in 30-day mortality and in-hospital stay, highlighting the timely intervention due to early detection by AI. Intensive care unit (ICU) transfers were also reduced (39). However, an increase in ICU stays was observed, possibly due to earlier identification of subtle signs by AI or due to over-reliance on AI by physicians, particularly if skeptical about their own diagnosis, leading to unnecessary admissions (39). Although predictive models help in timely detection and resource allocation, they may worsen existing inequities by favoring wealthier patients, and their lack of transparency in risk assessment limits clinicians' trust (40). Electronic health records (EHR) focus on the health of data of patients during doctor's visits and beyond. Combining AI tools with patient-generated health data (PGHD), such as lifestyle habits, environmental factors and behavior, which can be collected via AI wearable devices or apps, can sustain the continuous monitoring of the health of a patient. The integration of EHR along with PGHD can improve individualized patient care and treatment (41).

### Precision treatment

Integrating AI and genomic medicine holds promising value in identifying potential disease outbreaks via ML algorithms (42). AI monitors novel threats, such as COVID-19, whereas genomic data shed light on the susceptibility of certain groups to specific diseases that traditional methods, such as statistics, can often overlook (43). In order to predict genes linked to autism spectrum disorder, Krishnan *et al* (44) presented a ML, brain-specific gene-network technique that revealed network-level indicators, which are challenging to identify by manual inspection.

The fusion of genomic medicine with AI has made advancements in drug discovery through the usage of existing medications for new disease purposes or uncovering new targets for therapy (45). However, at present, its non-clinical toxicity still poses a challenge. It enables post-market drug withdrawal, as it is capable of predicting drug toxicities, such as cardiotoxicity and hepatotoxicity (46).

Technologies, such as ML algorithms use past and current medical information for developing predictive tools, which in turn assist in cost reduction, as well as in improved patient outcomes. The predictive tools are prominent in assessing the risk of developing chronic diseases, such as endocrine and cardiac disorders in patients (47). The tools take into consideration the medical history, demographics and lifestyle factors of patients, stratifying higher-risk group patients and aiming interventions to prevent or treat them. Another application of the predictive models is to stratify patients into groups that are at higher risk of hospital readmissions and focus on preventing them via interventions (47).

A data analytics tool in Saudi Arabia, known as Sehaa, can detect diseases based on Twitter data. The tool contributed to finding the top chronic diseases in one city and found the healthiest city. This highlights the potential of the predictive tools in the health management of populations and the urgent intervention required in Saudi Arabia for the prevention and treatment of chronic diseases (35). In addition, such examples highlight the need for governance and evaluation. Existing AI frameworks emphasize transparency, reproducibility, ethics, effectiveness and stakeholder engagement when deploying predictive analytics in medicine (48). AI algorithms also achieve improved results for patients. For instance, in their study, Barmaz and Ménard (49), used AI to observe how efficiently doctors reported safety issues. The tool aided in safety improvement via detecting problems early without the need for frequent inspections (49). Another study by Wang *et al* (50) concluded that AI could predict patients who are at risk of falling, thus preventing injuries.

Traditionally, clinicians rely on the trial-and-error method for prescribing medications, which is inefficient and may harm patients. AI can reduce this by matching the drug best suited for the patient or predicting errors (51). Corny *et al* (52) developed a system that alerted physicians about unsafe medications before prescribing and identified 74% of risky prescriptions. Zheng *et al* (53) found that doctors trusted AI more when it explained its medication decisions. Additionally, AI personalizes the dose and prevents side-effects. This was supported by a study that reported that clinicians were outperformed by algorithms in generating reliable individual warfarin doses (54). Lastly, an AI tool, known as CURATE AI, adjusted chemotherapy doses based on the responses of patients to treatment. It decreased the dose, increased the response and reduced the harmful effects (55).

## 4. AI in surgery and robotics

Surgery is being utilized with AI and robotics, which enhances patient outcomes, accuracy and efficacy. Healthcare systems are globally utilizing AI-driven approaches to address key challenges such as accessibility, minimally invasive procedures and surgical efficiency (56-58). AI integrates computational tools, such as ML, augmented reality (AR), computer vision and operational robotics, all of which improve surgical performance and clinical judgement (56,59,60).

### AI-assisted surgical planning

Surgical pathways are increasingly supported by technology, providing quantifiable markers to assess outcomes. This foundation aids the development and approval of AI-driven software-as-medical-device (SaMD) tools across the surgical workflow (61). These tools include automated surgical devices with AI support, radiomic systems for screening and diagnosis, models that employ clinical data for operational risk evaluation, and decision-support tools for postoperative care and monitoring (61). Additionally, several studies have found that diagnostic precision is either comparable to or superior to that of human specialists. Therefore, patient outcomes are improved by the ensuing advances in diagnostic speed and accuracy, which enable quicker and better surgical decision-making (62).

### Pre-operative risk prediction

Pre-operative risk assessment tools have become essential resources for patients and physicians to predict the possibility of surgical complications. The acute physiology, age, chronic health evaluation (APACHE) III prognostic system for critically ill patients, the Physiological and Operative Severity Score for the enUmeration of Mortality and morbidity (POSSUM) scoring system for predicting morbidity and mortality, and the American College of Surgeons National Surgical Quality Improvement Program (ACS NSQIP) surgical risk calculator are examples of commonly used models (61). Recent advances in AI, particularly ML, have made these technologies more reliable. Large, varied datasets can be processed by ML models, which can also identify intricate, non-linear relationships, leading to more dynamic and accurate risk categorization. Using the ML-derived classification tree, the Prediction Optimal Trees in Emergency Surgery Risk (POTTER) calculator increased prediction accuracy over the ACS NSQIP for patients undergoing emergency laparotomy. Similarly, using data from electronic health records (EHRs), the MyRiskSurgery algorithm, developed and validated by a study, helped predict major postoperative complications and mortality. This allowed for the real-time monitoring of evolving risk factors and continuous improvement based on clinical feedback. Future instruments for individualized risk assessment should incorporate a variety of patient, physician, and procedure-specific criteria (61,63). In addition, real-time AI tools (e.g., intraoperative video feedback, adaptive neuro-visual systems) can reduce surgical trauma and errors (64-66). During cardiac surgery, RNNs have proven to be more accurate than traditional methods in forecasting complications, such as bleeding, renal failure and mortality (67).

### Robotic surgery platforms

Robotic systems enhance surgical performance by improving dexterity, ergonomics and 3D magnification. Convolutional neural networks have been used in surgical training to standardize evaluations and reduce bias by objectively assessing basic technical abilities such as suturing and knotting (61,68,69). Even though existing robotic platforms such as the da Vinci system are still directly controlled by surgeons, future autonomous systems may enable fully independent treatments, providing precision, fatigue-free performance and possible applicability in distant or high-risk areas (69-71)

### Intraoperative AI use

DL techniques, such as semantic segmentation show promise for intraoperative guidance, identifying safe vs. hazardous regions (as in laparoscopic cholecystectomy) (72). Similar techniques are being studied in other procedures such as sleeve gastrectomy, cataract surgery and aneurysm repair (61,73,74). AI-driven 3D reconstruction and navigation also aid surgery; for instance, the FDA-approved Cydar EV Maps tool aligns pre-operative imaging with real-time fluoroscopy in aneurysm repair (75). AR enhances intraoperative vision by overlaying semitransparent preoperative images onto the surgical field. One approach employed 3D contour matching to align virtual images with real teeth during oral and maxillofacial surgery, while another projected a 3D vascular model onto the lower limbs of a patient using the HoloLens. Additionally, 2D and 3D eye-gaze tracking facilitates robotic surgery by improving navigation and instrument control. Eye-gaze contingent devices enhance guidance, precision, and image transmission. Gesture recognition is also crucial for human-robot interaction since technologies such as Gestonurse and Hidden Markov Model-based techniques facilitate tool delivery and AR control (66).

### Post-operative outcomes

Robotic-assisted surgery and AI have exhibited peri-operative benefits: Reduced blood loss, fewer transfusions, shorter periods of hospitalization and fewer complications (61). A 2024 meta-analysis comparing open or laparopelvic surgery to abdominopelvic robotic surgery revealed that abdominopelvic robotic surgery was associated with a significantly shorter period of hospitalization (mean difference, -0.23 days, P=0.01 vs. laparoscopy, -1.69 days, P=0.001 vs. open) and a significantly lower estimated blood loss (mean difference, -286.8 ml vs. open) (76). AI can further improve outcomes by predicting post-operative risks. The model successfully identified high-risk cases and suggested whether the usage of defunctioning stomas was advisable or not (77,78). Additionally, wearable and implanted sensors enhance post-operative care by promoting early discharge and enhancing problem identification (66). Furthermore, Shahi *et al*. demonstrated how incorporating AI into robotic surgery improves functional recovery and reduces post-operative complications by enabling real-time feedback and error correction (79).

### Limitations and safety concerns

AI integration in surgery requires cautious, ethical planning (61). Due to their high acquisition and maintenance costs, AI and robotic systems are out of reach for a number of public and rural healthcare settings (56,80). Logistical challenges also arise due to complex system configuration and the need for highly experienced staff, particularly in settings with limited resources (59,81). The limited capacity of AI algorithms to adjust in real-time to anatomical changes and inconsistent results raises concerns since they may erode surgeon confidence (60,82). Digital twins and real-time image guidance are examples of emerging techniques that rely on trustworthy, high-fidelity data that are not yet generally accessible. Integration into clinical operations is further complicated by incompatibilities between various robotic platforms, AI software and hospital IT systems (83-85).

Based on current research, surgical systems assisted by AI and robots are associated with markedly reduced intraoperative blood loss and shorter periods of hospitalization compared to traditional approaches. For example, periods of hospitalization were shortened and anticipated blood loss was decreased in the case of robot-navigated pedicle screw insertion (86). Fully autonomous surgical systems, which take the surgeon out of the control loop, are still in the early stages of clinical translation (69,87).

*Barriers and pragmatic solutions for low-resource hospitals.* There are various barriers hindering the successful use of AI tools in hospitals with limited resources, such as:

*i) Limited infrastructure*. No high-speed networks, cloud computing or AI-based surgical tools (88). Inadequate infrastructure and high implementation costs remain major obstacles. The majority of institutions in low and middle income countries (LMICs) cannot afford the high expenses of robotic consoles, maintenance and specialist training, despite the fact that robotic surgery is becoming a standard component of minimally invasive operations in high-income countries (89).

*ii) Data gaps and insufficient local adaptation*. The absence of high-quality, regionally relevant surgical datasets limits the accuracy and applicability of the model (88).

*iii) Lack of AI expertise*. The absence of AI training among healthcare workers impedes the adoption of AI (88).

*iv) Policy and financial constraints*. High training and technology costs, along with insufficient or non-existent regulatory frameworks are also a barrier (88).

Recommendations include the improvement of capacity through collaborations with educational and technology institutions to train medical professionals in AI and ML (88). Strengthening public-corporate relationships between governments, international organizations and the commercial sector can help advance and grow AI-based surgical initiatives (88). AI models trained on LMIC-specific data need to be designed in order to improve diagnosis accuracy and contextual applicability (88). Enhancing digital health infrastructure is crucial to enable AI technologies, remote surgery and telemonitoring, in addition to implementing structured technological systems that include real-time translation, AI-driven performance analytics and reliable bidirectional communication. By establishing robust ethical and policy frameworks, the ethical, equitable and sustainable use of AI in surgical care may be guaranteed (88,89). Moreover, developing cost-effective AI-assisted surgical tools suitable for LMIC resource constraints remains a primary objective (88). Finally, the high capacity and ultra-low latency of 5G networks render them suitable for real-time telesurgery and immersive remote surgical training (89).

### Ethical and practical considerations

There are some ethical and practical concerns associated with the use of AI in surgery that need to be addressed. Error accountability is ambiguous, and there are security and privacy concerns when using patient data. Equitable access is a concern in areas with limited infrastructure (90). Reliance on algorithms can undermine trust between patient and surgeon, particularly in high-risk or pediatric cases where human judgment is essential. The effectiveness and integrity of treatment may be compromised by biases in AI training data, which are typically obtained from homogeneous groups (85,91).

## 5. AI in cardiology

Cardiovascular disease is the leading cause of mortality worldwide, rendering it a critical focus for innovation using AI. These chronic diseases require essential monitoring following acute treatment, and AI-based solutions are ideal for them (92). The AI-integrated remote patient monitoring (RPM) provides live data from cardiac implantable electronic devices, rendering it highly useful for timely diagnosis, early intervention and personalized care for patients (93).

The diagnostic abilities of AI are notable in the interpretation of results from electrocardiogram (ECG), echocardiography and CT angiography. The study by Siranart *et al* (94) demonstrated that ECG interpretation using deep learning models identified left ventricular hypertrophy with higher sensitivity and specificity in comparison to Sokolow-Lyon and Cornell criteria. A total sample size of 66,479 participants (with and without left ventricular hypertrophy) was part of that study which exhibited a pool sensitivity and specificity of 69% (95% CI, 47-85%) and 87% (95% CI, 76-94%), respectively for AI-based interpretation (94). Similarly, a randomized, blinded non-inferiority clinical trial of the initial evaluation of echocardiography assessment by AI and a sonographer provided clear insight into how workflow time and results were performed efficiently using AI (95). In that study, which evaluated 3,769 echocardiographic studies, a substantial assessment error of 5% from the initial assessment to final cardiologist assessment was observed. However, the substantial assessment of left ventricular ejection fraction was 16.8% (n=292/1,740 studies) in AI, whereas 27.2% (n=478/1,755 studies) by sonographers with a difference of only -10.4% between the groups (95% CI, -13.2 to -7.7%, P<0.001 for non-inferiority, P<0.001 for superiority) (95). An AI-enabled quantitative coronary computed tomographic angiography (AI-QCT) is a novel technique that uses convolutional neural network models for assessing the severity of stenosis and various atherosclerotic plaque components (96). With automated analysis by a software platform (Cleerly Inc.), AI-QCT provides coronary artery wall and lumen specification alongside the quantification of plaques (96). The AI-QCT also identified significant differences in atherosclerotic plaque based on age among the 303 patients in the CREDENCE cohort (96). In another study, in-hospital cardiac mortality due to acute heart failure (HF) cases was predicted more effectively using the Quantitative ECG (QCG), an AI-based ECG analyzer that uses printed ECG images (97). The QCG-Critical score among the 1,254 patients with acute HF in a prospective cohort study was higher in patients experienced in-hospital cardiac mortality than in survivors, with means of 0.57 (SD 0.23) and 0.29 (SD 0.20), respectively (P<.001). The strong predictive value of the AI-based score was demonstrated with an AUC of 0.821 for in-hospital cardiac mortality (97).

In the new era of smartwatches and digital stethoscopes, the medical usage of this technology in cardiology has also improved. A smartwatch ECG platform (Cardiologs^®^) enabled QT prolongation detection and premature ventricular contractions, with 98.2% of patients only exhibiting a <50 m/sec difference with a manual 12-lead QTc measurement (98). For rural and remote settings with lower resources or fewer cardiologists, a digital stethoscope developed by Eko Health helps in reducing the burden by simultaneously recording using 3-lead ECG and analyzing atrial fibrillation or murmurs (99). As the digital stethoscope can also share data, virtual cardiology consultations with specialists are possible, providing prompt decision-making according to the case, even from remote locations (99).

Early predictions of cardiovascular events are greatly supported by AI innovations. The major adverse cardiac event can be predicted using the cardiovascular magnetic resonance cine sequences with the aid of an automated AI-based assessment of cardiac deformation (100). The predictions by AI can be made using electrical features, such as heart rate variability, intervals of QT and QRS (101). The AI-trained models used to identify patients at risk of sudden cardiac mortality perform more effectively than the electrical risk scores from extracted electrical features, with a concordance index of 0.9, over the 0.74 for the electrical risk score (101). For patients using RPM systems, clinicians are provided with a lead time with the aid of algorithms that predict arrhythmia worsening or device issues prior to the onset of symptoms and therefore, prevent hospitalization (93).

The involvement of AI in optimizing treatment plans and the management of patients with cardiac events is found to be effective. AI-based quantitative coronary angiography (AI-QCA) used in the FLASH trial (among 400 patients) achieved the automatic analysis of angiography images during the percutaneous coronary intervention, aiding clinicians to select optimal stent and balloon sizing without requiring additional inputs or further processing time (102). Yet another implementation of a deep neural network was on the dosage prediction of warfarin for patients on the 5th day, predicted more accurately than physicians, by using RNNs to create a warfarin dose to the prothrombin time-international normalized ratio response over the results from the first 4 days of doses (103). Similarly, AI systems can support the guideline-directed medical therapy optimization of medications in patients with HF by predicting health deterioration in patients after analyzing the data derived from the wearable tools (104).

Furthermore, for patients in intensive care and those with persistent cardiac conditions, AI-assisted tools have proven useful. An FDA-approved ultrasound system named Caption Guidance was well used in patients with COVID-19 in the ICU, where it assisted critical care physicians with no formal ultrasound training to obtain cardiac ultrasound images from optimal views (105). Deisenhofer *et al* (106) conducted a large-scale, randomized study on 374 patients, that demonstrated the advantages of employing AI-analyzed intracardiac electrograms to direct catheter ablation in patients with persistent atrial fibrillation. Similarly, an augmented intelligence named EchoGo Core by Ultromics, cleared under FDA 510(k) K191171(107), uses AI technology to create accurate and reproducible cardiac measurements, such as ejection fraction and left ventricular volumes (108). The triage platform by Viz.ai can aid with risk stratification by prioritizing suspected pulmonary embolism, with AI tools also supporting automated CT-derived RV/LV ratio assessments (109); the FDA-approved device is Viz RV/LV (K221100) (110). In addition, the BriefCase-Triage system (K251406) by Aidoc (110) facilitates the rapid prioritization of suspected aortic dissection (111). There is a clear clinical shift in the usage of AI with other FDA-approved systems for coronary artery disease diagnosis using HeartFlow FFRcT Analysis (K190925) (110,112). Other recent FDA-cleared cardiovascular AI tools include DeepRhythmAI (K250932), InVision Precision Cardiac Amyloid (K243866), Volta AF-Xplorer (K243812), AT-Patch (ATP-C130/ATP-C70) (K242583), VitalRhythm (K242129) and the Loss Of Pulse Detection (K242967) by Fitbit (110). Rapid advancements in AI in cardiology are facilitating more accurate and tailored clinical judgments; these advancements hold promise for improved patient outcomes in the future. A brief summary of AI applications per specialty is provided in Table SII.

## 6. Benefits and opportunities of AI in clinical practice

Healthcare services in this era face numerous challenges, such as an increasing number of patients requiring advanced medical needs, and a shortage of medical supplies. AI provides a high-impact system that increases productivity, improves clinical outcomes and hospital workflows (113).

### AI in diagnostic accuracy

Incorporating AI into medical operations has notably increased identification accuracy and diagnostic accuracy (113). AI evaluates extensive clinical data more efficiently, recognizing fine details that may be missed on conventional analysis. In radiology and pathology in particular, which require specialized expertise and large data handling for lesion detection and bone metastasis, AI has aided in reducing the diagnostic and workload times by ~90% (114). AI tools assist in the timely identification of clinical cases such as diabetic retinopathy and systemic infections, as well as the metastasis of cancer, enabling prompt intervention for improved prognosis (115). AI tools also aid in the recognition of periodontal problems by scan interpretation and in evaluating neurobehavioral disorders, including stress assessment (116).

AI executes operations such as lesion spotting, morphological analysis and computerized image analysis, allowing medical professionals to prioritize critical decisions (114). It minimizes human errors and ensures patient safety by providing an additional perspective. Low-quality or prejudiced data may also yield misleading conclusions, emphasizing the necessity for comprehensive data management and transparent data sourcing (115,117).

### AI in workflow enhancement

Language-based tools are often used for digitizing medical records, including patient encounter records to update EHR. This eliminates the paperwork and strengthens record accuracy. AI can allocate efficient schedules based on staff availability and care requirements. A previous study demonstrated a 12% decrease in extended-hour wages (118). AI-enabled virtual assistance manages routine patient inquiries, schedules consultations, and triages them, enabling staff to focus on specialized intervention (118,119).

*Enhancing personalized medicine with AI*. AI is critical for interpreting comprehensive patient data and can generate treatment plans customized to individual care. This approach boosts success, shifting away from conventional generalized care (113). Generative AI spots patterns to customize therapies; it can anticipate cardiovascular risk from ocular visuals and identify biomarkers for complex diseases. AI algorithms such as GENTRL assist in therapeutic formulation, which targets specific biological mechanisms. AI can forecast individual responses to a drug, therefore giving customized prescriptions (120). AI facilitates the rapid analysis of DNA profiles linking genetic variations to risk associated with the disease and treatment responses (115). Gen AI drives software adaptation for personalized tools such as CBT in psychological care (120). The progress of advancement depends on the availability of high-quality patient data. Ensuring informed consent about data privacy is crucial for enabling trust in AI systems (119).

### Strengthening early detection and prevention

AI strengthens preemptive discovery and the avoidance analysis of large, diverse data sets to pinpoint structures and predict risks (121). AI automated Early warning systems can recognize contagious disease surges more rapidly than conventional methods by interpreting records from epidemiology, environment and online trades. Software tools such as BlueDot can detect the preliminary symptoms of COVID-19(121). These tools can also anticipate clinical risk, including re-hospitalization and deterioration, using medical records (118). In community health, AI can forecast disease development based on biomarkers and social markers (120). Biosensors combined with AI enable live health monitors and early prediction of chronic conditions, including diabetes and heart disease. AI supports mental health by screening and symptom tracking of diseases like dementia through speech evaluation (115). This system aids in pandemic planning by the deployment of healthcare supplies (121).

### Driving cost reduction in healthcare

AI can aid in reducing costs and optimizing productivity in both administrative and therapeutic areas (122). By digitizing tasks such as billing and insurance, AI can conserve up to $168 billion yearly in operational costs (122). AI assists clinical professionals make evidence-based decisions by limiting inefficient procedures and tests. A recent study documented the completion of >45,000 tests within only 45 days. By anticipating patient requirements and disease, AI advances in resource allocation assist hospitals in managing beds and supplies (123). AI tools can also identify malpractice. Recent data from IBM highlights a successful recovery of >$41.5 million in Medicaid funds (115). Moreover, the integration of AI fosters enhanced financial optimization by increasing transparency and minimizing treatment fragmentation (122).

### Revolutionizing training and education

AI is revolutionizing medical training and professional development by granting them training and upskilling (120). AI-based simulations provide human-like cases that help students to practice clinical skills in a risk-free setting (119). In surgical training, AI provides instant evaluation on skill assessment. These innovations reconcile conceptual and practical knowledge (120). Modern medical care emphasizes trust and ethical development through AI-assisted teamwork (113,115,120).

## 7. Challenges and limitations

### Data bias

The most prominent ethical concern in AI in healthcare is bias in data sources, that is, the data used to train AI systems is not neutral, but instead is frequently biased based on certain factors, such as sex, race, socioeconomic status, geography, or sexual orientation (124). A study conducted in the USA observed that doctors disregarded positive AI predictions for African Americans due to a high false positive frequency. This occurred due to insufficient data related to them in the AI training system. Hence, the model was inaccurate, leading to unreliable and biased outcomes (125). AI can amplify the existing bias based on historical data. It can reinforce subjective biases when it learns in history that the clinicians opt not to treat patients with extreme preterm birth or brain injury, thereby placing the lives of patients at risk (126,127). Additionally, AI can serve the wealthier patients more efficiently than the patients from less wealthy communities, as it notes the latter may overstay in the hospital due to no transport or support at home, allocating them a lower priority (128). AI can pose a serious threat in the event that there is no expert oversight, hence introducing automation bias (129).

### Black box error

The complex nature of algorithms through which AI systems reach conclusions is not yet fully understood or interpreted by the physicians who use these tools, and this becomes a serious concern when AI contradicts clinical logic (130,131). It has been reported that AI rated patients with pneumonia and co-existing asthma as low-risk, while it incorrectly flagged patients with pneumonia alone as high-risk. This error was likely due to the misinterpretation of data; however, to the physicians using the tool, it remained unclear. Instances such as these render clinicians skeptical of fully integrating AI tools into daily practice (132).

### Mitigation strategies for bias

To reduce bias and improve transparency, AI tools need to be audited regularly before and after being used in the hospitals. Developers also need to disclose the details about demographics used to train the model to detect an imbalance early. Moreover, testing the system on patients in other clinical settings examines the reliability of the model (133).

### Ethical and legal issues

At present, there are no global laws or standards defining who is responsible, whether it is the developer, user (physician), or system maintainer when AI leads to a harmful outcome. This can subsequently lead to unfair blame on physicians even when decisions were heavily AI-influenced (134). Data ethics, an understructure of AI, includes informed consent, privacy and data protection, ownership, objectivity and transparency. Controversial data sharing is highlighted by cases such as DeepMind and the Royal Free London Foundation Trust. Although patients in Canada are the legal owners of their data, hospitals act as custodians, which poses questions around rights and transparency (135). The recent increase in the number of individuals receiving genetic testing emphasizes the urgent need for stronger privacy laws, given that genetic data is sensitive information that could be used for biowarfare or discrimination (136).

### Regulatory uncertainty

Laws such as the Health Insurance Portability and Accountability Act (HIPAA) were created in the 1990s to protect patient data, long before AI became essential in healthcare. However, this is inadequate in the present time, particularly with the complexities of modern AI tools (137). Furthermore, McCartney (138) noted that the NHS 111 algorithms had undergone user testing and that a Babylon-powered NHS 111 pilot was underway; however, at the time there were few formal, published clinical evaluations assessing its safety or effectiveness. On that account, reform needs to be made, such as updated laws, new regulatory bodies, and a clear division of responsibility among developers, users and maintainers (139).

### Overreliance and de-skilling

When clinicians become overly reliant on AI systems, it results in a decline in critical thinking and decision-making skills, consequently contributing to de-skilling. This is supported by research that has observed a decrease in diagnostic accuracy in physicians relying heavily on AI systems, such as automated image analysis or computer annotations (140). Overreliance on AI may set back the development of necessary surgery skills, particularly in fields such as neurosurgery (140).

### System integration and usability

The integration of AI into hospitals and combining different systems remains a notable challenge. While tools such as the European eHealth Interoperability Framework (eEIF) and API-based integration approaches have been introduced to aid healthcare providers securely and efficiently share patient data, they are far from perfect. These systems are often associated with high costs, technical difficulties, concerns around patient privacy, and issues related to scaling and performance (141). Additionally, usability persists as another challenge. The busy workflow of physicians, along with difficulties in understanding or navigating the AI tools, may likely discourage them from using the tools. Each hospital uses a different electronic system, and hence the AI tools need to be specifically adjusted, making it more difficult to implement across various healthcare settings without sufficient work placed into its setup (141).

## 8. Ethical and legal considerations

For the ethical deployment of AI in medical care, physicians need to respect patient autonomy and obtain valid consent prior to decision-making. It is essential to keep the patients aware of the extent of AI involvement and its role in their diagnosis or therapeutic process, as technology is increasingly assisting in clinical decisions (142). The integration of AI into healthcare also raises concerns regarding various ethical principles such as the following:

### Patient autonomy and informed consent

Clinicians should retain meaningful control, including the ability to explain (and, if necessary, override) AI-generated suggestions, and patients should be informed when AI-based decision-support systems are employed in their treatment (143). This aligns with recommendations that consent forms explicitly disclose when AI is used and explain how the AI system functions and its risks to the patient (144).

### Data privacy and security

AI depends on large volumes of sensitive medical information therefore it's essential to protect patient confidentiality (145). As a result, AI systems require robust data-protection safeguards, such as, de-identification, secure storage, appropriate access controls, and continuous security monitoring. They also need to adhere to relevant regulatory frameworks, such as General Data Protection Regulation (GDPR) and HIPAA (144).

### Algorithmic bias and fairness

In the event that the tool is not trained on a dataset that is representative of a whole population, it may lead to biased results (146). Experts emphasize training on diverse, inclusive datasets and conducting ongoing bias audits; for example, the continuous monitoring of model performance across demographic subgroups can identify and correct emerging biases (144,147). Regular monitoring and evaluations of the algorithms provides fair and accurate results across different patient groups (146).

A breakdown in informed consent occurs when patients are unaware of how their health data are used or stored, leading to mistrust and privacy concerns due to missing details in consent forms (148). AI systems such as Watson for Oncology raise concerns of ‘machine paternalism’, where preset parameters override patient preferences (149). To mitigate this issue of autonomy compromising, the patients should be notified prior to the involvement of an AI device in the diagnosis or treatment and must be given the choice of accepting or rejecting it (150). Dynamic consent models involving ongoing patient engagement and transparency about AI use have been proposed to further safeguard autonomy in AI-driven care (144). This approach ensures that patients can opt for second opinions or human alternatives, as they are adequately informed regarding the role and limitations of AI tools (142).

Due to the differences in the systems of healthcare datasets, bias and equity are integral in AI ethical assessment. The AI models often underperform in marginalized populations as their training is mainly on unrepresentative datasets. In a previous study, an AI-based melanoma model provided poor predictions and a delay in diagnosis on darker-skinned individuals, as their training was primarily on light skin tones (151). Incomplete records and the exclusion of social determinants among marginalized groups contribute to biased AI performance. The lack of diversity among developers can also pose a challenge, as unconscious preference for perspectives may occur (152). Certain tools such as Shapley Additive Explanations (SHAP) and Local Interpretable Model-agnostic Explanations (LIME) can help to align with equity principles by revealing hidden biases (151). Experts also emphasize the need for Diversity, Equity and Inclusion (DEI) principles throughout the AI development lifecycle, including educating and training a more diverse developer workforce and structured bias mitigation programs (147). Ultimately, it is essential to identify further methods to eradicate biases to ensure the smooth functioning of AI. A legal and ethical ambiguity exists in the accountability for the decisions made by AI. For situations where an erroneous diagnosis is made by AI, it is unclear who among the developers, clinicians, or institutions will be held responsible (153). Such complex situations are difficult to address with traditional legalities, as AI tools are variable and evolving faster (154). Indeed, a previous review observed that when AI errors occur, it is unclear who is accountable, the physician utilizing the AI, the organization putting the technology into practice, or the developers of the algorithm (144). The algorithm developers escape the scrutiny, and doctors are often held accountable legally (154). Recent analyses note that while some argue clinicians need to retain ultimate responsibility, others suggest that developers and regulators should also share accountability, highlighting the need for clear multi-stakeholder liability frameworks (155). To maintain professional responsibility, it is essential to have systems to report incidents and a clear delineation of roles (156). The duties for AI developers, clinical sponsors and other stakeholders, including physicians, are proposed by the Medical Device Regulation (MDR) and the European Artificial Intelligence Act (AIA) (157). For example, the AI/ML-based medical device framework proposed by the FDA emphasizes ongoing real-world performance monitoring and explicit role definitions for clinicians and developers to ensure accountability (147).

One of the primary ethical concerns that remains with the use of AI involves data breaches and lapses in security. The sensitive personal data of patients, including their demographics, genomics and medical histories, is largely used by the AI systems (153). As data are sourced from multiple contact points and then distributed over various platforms, it is susceptible to breaches (153). As medical AI technologies are becoming more commercial, the exploitation of data and potential re-identification of hidden data occurs (153). The HIPAA in the USA and the General Data Protection Regulation (GDPR) in Europe mandate strict regulation in consent management, anonymity and access controls (35,142,158). The MedRec, a blockchain technology, uses cryptographic hashes and smart contracts that enable controlled access to the medical data, which aligns with GDPR Article 9 and HIPAA 45 CFR (Code of Federal Regulations) (158). Researchers also promote privacy-by-design approaches: For instance, federated learning allows models to train on decentralized local data without sharing raw patient records, thus preserving privacy (147). The cyber threat can be detected by using predictive algorithms, and for ethical sharing of data, federated learning is promoted (35,156).

Transparency is key for participatory decision-making in AI-based healthcare. The ‘black box’ nature of AI tools often makes it difficult for patients and clinicians to understand the decisions made by them (154,159). The professional responsibilities and informed consent are hindered by this opaque functioning of AI (154). To achieve transparency in an AI system, various measures can be implemented, such as communication, traceability, interpretability and auditability along with robust data management (150). It is advised that developers embed explainable AI that will help users understand medical context (148,156). In practice, this means that patients should be explicitly informed about how the AI works and its limitations during the consent process (144). Such a feature allows individuals the right to understand automated decisions made by AI, and this aligns with the GDPR mandates (148).

The existing regulatory frameworks are behind the fast-paced evolution in AI, exposing the gaps in the various global standard frameworks. The DermAssist, an algorithm mainly trained in light-skinned individuals, bypassed stringent trials and was self-certified in the European Union (EU) as a Class-I device irrespective of its undermined reliability on darker skin tones (149). A previous study indicated that ethnic data or socioeconomic strata are only provided among a few of the FDA-approved devices, which may cause hindrance in fair assessments (160). The regulations on AI approval have discrepancies across the globe, even among bodies such as the FDA and the European Medicines Agency (EMA) (157,159). The World Health Organization (WHO) has issued guidelines advising on a transparent, accountable, and inclusive framework, yet a universal legal framework is absent (153). A recent review warns that, without standardized oversight, AI systems trained on biased or insufficiently representative datasets may underperform for underrepresented populations and thereby exacerbate healthcare inequities (144). The authors of that review recommend regulatory standards requiring inclusive data, external testing and oversight to mitigate these risks (144).

In conclusion, at the institution level, the authorities can have a formalized series of guidelines to be followed before using AI to protect patient rights, clinical accountability and compliance with regulatory boards. Patients need to be adequately informed regarding the procedures in which the AI assistance is used and their explicit informed consent in accordance with GDPR/HIPAA privacy standards. The clinical validation of data needs to be carried out routinely, and the regulatory status of the device or software should be documented at regular intervals. In addition to the AI system results, a human override should be ensured with a defined protocol, which can help in retention of authority of final decision making with experienced clinicians in high-risk scenarios. The results reported by the AI mechanism can be integrated with existing medical records to capture and act promptly in cases of system errors and model drifts. Staff training is crucial: Clinicians and support staff need both technical proficiency with AI systems and the communication skills to explain the limits of AI to patients, document consent and recognize when human judgment must prevail.

Best-practice frameworks such as Developmental and Exploratory Clinical Investigation of Decision support systems driven by Artificial Intelligence (DECIDE-AI) recommend the phased clinical testing and ‘shadow deployment’ of AI tools to identify biases and safety issues before full rollout (147). As AI continues to provide transformative ability in the field of medicine, a coordinated legal and ethical framework is vital to safeguard the patients. An overview of the regulatory frameworks and guidelines for AI in healthcare by region is provided in Table SIII.

## 9. Future directions and innovations

AI continues to transform the healthcare landscape with notable advancements in imaging, statistical analysis and surgical robotics, improving procedure efficiency and patient safety. The use of AI in precision medicine, imaging, diagnosis, risk stratification, genomics and drug discovery is rising (56,161).

### AI in imaging and screening

AI is increasingly being employed in image-based screening tools. Lesion detection AI tools, such as Transpara, INSIGHT MMG and ProFound have demonstrated promising outcomes in screening mammography. Prospective, population-based trials of Transpara and INSIGHT MMG, published in 2023, have reported encouraging findings, suggesting that deep learning systems may be used as reliable second readers in the future, which would reduce the workload of radiologists and improve the cancer diagnosis rate (162,163). Similar approaches are being researched for colorectal and lung cancer screening (164,165).

### Workflow optimization

AI-driven workflow triage systems appear to have a promising future in clinical data stratification by risk category. While high-risk cases can be prioritized for an earlier physician assessment, low-risk data can be processed more efficiently, and this approach is especially beneficial for managing the huge amounts of data generated by routine screening and diagnostic processes (166).

### Integration with multiomics

Molecular biology is undergoing a paradigm shift due to the application of multiomics approaches, which analyze large datasets comprising the genome, epigenome, proteome, metabolome and microbiome. The application of AI in multiomics is still in its early stages; however, by integrating genomic, imaging and clinical data, it could enhance the identification of at-risk groups, and enable more precise screening, diagnosis and prognosis (167,168).

*Data collection and standards*. Multiomics datasets need to be collected under standardized protocols and common data formats to ensure consistency and quality (169). Establishing clear standards (e.g., FAIR data principles) and rigorous data curation will ensure that the datasets are robust and interoperable (170).

*Harmonization and federated learning*. Implement distributed learning architectures will allow institutions to train shared models without exchanging raw data. Data harmonization and federated learning can integrate diverse genomic, proteomic and clinical data across centers, while preserving patient privacy (171).

*Model development and external validation*. Predictive models using a range of multiomics cohorts that are representative of the population could be established and these could be rigorously tested on external datasets. External validation across distinct populations is crucial for avoiding overfitting and ensuring generalizability. By using transfer learning and domain adaptation strategies, model resilience across different patient groups can be further improved (169).

*Regulatory and deployment considerations*. Developers should incorporate regulatory planning early in the development process and adhere to evolving frameworks, such as guidelines on AI/ML-based medical devices, to ensure transparency, patient safety and clinical implementation that aligns with regulations (157,172).

*Monitoring and maintenance*. Following installation, AI/ML systems need to be subjected to regular observation to detect drift or bias. Performance updates and issue reports need to be made on a regular basis. The EU AI Act and other legal frameworks require high-risk AI systems to maintain these supervision obligations for the duration of their functioning (173,174).

### Advanced diagnostic tools

Rong *et al* (175) reported that AI applications have been used to support diagnosis, disease management and clinical decision-making across several areas of medicine, and thus may improve diagnostic accuracy and workflows. NLP and automated voice recognition technologies have the potential to enhance clinical documentation by condensing crucial information from doctor-patient interactions (176).

### Mixed reality and intraoperative guidance

As interest in mixed reality technologies, such as virtual reality (VR) and AR increases, so does intraoperative guiding and surgical planning. VR immerses users in a virtual world, whereas AR overlays the operator's field of vision with digital graphics in the actual world. The future integration of AI and AR could provide dynamic, immersive surgical guidance and real-time 3D anatomical reconstruction (61,177).

### Surgical training and simulation

AI and ML can enhance VR and AR-based simulation platforms, opening up new possibilities for surgical training. With the ability to address difficult learning curves in complex surgical specialties and to facilitate skill acquisition through customized and adaptable simulation settings, these technologies hold promise as beneficial teaching aids (61). Future advancements in robotics, bioprinting and personalized surgery could involve intelligent micro- and nanorobots for medication delivery and noninvasive medical procedures. Pre-operative evaluation data can be used for robotic assistance in the manufacture of 3D-bioprinted organs and tissues tailored for each patient and could advance transplant surgeries (66).

### Emerging challenges and research needs

Future research is required to focus on improving the accuracy, dependability and transparency of AI models in real-time clinical contexts (178,179). Further longitudinal studies are also required to evaluate patient outcomes and overall cost-effectiveness (80,180). Research into digital twins, extended reality integration and semi-autonomous robotic systems is likely to improve preoperative planning and simulation (83,179,181). A summary of future AI technologies and expected timelines for clinical implementation is provided in Table SIV. The ethical considerations of algorithmic responsibility, patient consent and equitable access to technology must remain at the forefront of the development and use of AI in healthcare (180,182). The effective integration of autonomous robotic-assisted surgery into clinical practice requires collaboration between the surgeon and the manufacturer to explicitly define duties, particularly in cases where malfunctions potentially endanger patient safety (82). A summary of the AI tools and systems discussed in the present review is provided in Table SV.

### AI in Healthcare: Anticipated advances in the ensuing 5-10 years. Education

Over the following decade, competence in AI will become increasingly critical in clinical training. Surveys indicate that staff and students are eager expand their knowledge of the use of AI in healthcare. For example, one survey found that only 50% of teachers and 30% of students felt acquainted with AI notions, despite their considerable interest for additional training (183). A different recent survey found that almost three-quarters of medical students supported structured AI instruction in their curriculum, with a focus on decision-support tools, ethics and applications (184). Future educational programs will emphasize the ethical use of AI, bias awareness and the critical evaluation of model outputs, preparing doctors to collaborate with AI-enabled systems rather than be replaced by them (183,184).

*Regulation*. Over the coming decade, continuous, evidence-based oversight is expected to become the norm for surgical AI control. The FDA is moving toward a lifecycle regulatory strategy for AI/ML-based surgical devices that emphasizes transparency, post-market evaluation and iterative performance monitoring to ensure safety and reliability in real-world use (185). In order to minimize bias and misuse in surgical AI applications, larger ethical and regulatory studies are required to further emphasize the importance of governance structures that include risk management, accountability and interdisciplinary stakeholder interaction (157).

*Reimbursement*. Health systems will expand payment methods to incorporate AI-driven surgeries. For example, studies on AI systems that produce current procedural terminology codes from operating notes indicate emerging reimbursement prospects in surgical specialties (186). A previous review of the broader AI/medical-device ecosystem demonstrated how reimbursement systems are evolving in parallel with regulatory monitoring, underscoring the necessity for coverage plans that keep up with innovation (187). Future government payers and insurers are expected to cover more validated AI tools in the surgical context, which will make integration viable and sustainable (186,187).

*Pilot trials*. In the coming years, phased pilot deployments of AI in clinical practice will expand its scope of application. Early implementations of ambient AI scribe technology have exhibited improved documentation efficiency, less labor after hours and a reduction in the workload of clinicians. Through iterative evaluation and refinement, these pilots will provide a scalable approach for more widespread AI integration, while ensuring usability and safety (188,189).

## 10. Conclusion

AI has been a transformative force within contemporary clinical practice, exhibiting stellar performance across the diagnosis, treatment planning, surgery and cardiology spaces. It also provides advanced decision-support and predictive analytics tools that leverage large-scale clinical data for more precise interventions. DL and other related technologies have notably improved imaging interpretation and lesion detection, and systems grounded in AI aid decision-making and permit more accurate therapeutic interventions. These capabilities can also automate routine tasks and prioritize urgent cases, optimizing clinical workflows. Within surgery, AI-assisted planning, intraoperative navigation and robotics have increased accuracy and efficiency, and have improved patient outcomese. In the same manner, within cardiology, ML algorithms have improved electrocardiographic and imaging interpretation, enabled remote monitoring, and enabled interventions that are often superior to those derived from traditional methods. Moreover, AI-powered wearable devices and digital tools (such as smartwatches and digital stethoscopes) extend cardiac monitoring to remote or underserved regions, enabling continuous care and virtual consultations. These inventions taken together highlight the capacity of AI to provide elevated diagnostic accuracy, streamline processes and individualize patient care. Notably, by reducing errors and streamlining workflows, these AI advances also support the healthcare ‘quadruple aim’ of improved outcomes, enhanced patient experience, lower costs and higher provider satisfaction.

Concurrently, key obstacles mitigate this advancement. Data bias continues to be a prevalent issue, posing the risk of reinforcing disparities in healthcare provision. The opaque ‘black box’ characteristics inherent in numerous models prompt inquiries regarding interpretability and trustworthiness, while unresolved ethical and legal challenges, such as liability, informed consent and data privacy, necessitate immediate focus. Current regulatory structures have failed to evolve alongside the swift technological progress, and the dangers of excessive dependence on AI underscore the necessity of maintaining critical clinical competencies. Furthermore, extensive training and governance are essential to ensure that clinicians remain proficient and can effectively oversee AI systems in high-risk scenarios. Additionally, challenges related to the incorporation of AI into current hospital infrastructures and operational processes persist, obstructing broad acceptance.

Despite these hurdles however, the potential for AI in healthcare is indisputable. Through improved diagnostic precision, the capability for enabling personalized therapies and improved efficiency, AI can lift the quality and value of healthcare into the future. Ensuring that such potential is fulfilled will require adequate validation, transparent and equitable development, and cross-disciplinary collaboration. Through adequate governance and sustained advances, AI can transcend the status of adjunct tool and become the cornerstone upon which medicine in the contemporary era is constructed, reconfiguring clinical practice for the benefit of patients globally.

## Supplementary Material

Comparison of the performance AI vs. that of human experts across different specialties.

Key clinical applications of AI by specialty.

Regulatory frameworks and guidelines for AI in healthcare by region.

Future AI technologies and expected timeline for clinical implementation.

AI tools and systems in contemporary medicine

## Data Availability

Not applicable.
