# Supplementary material for: Artificial intelligence in modern clinical practice (Review)
Source: Med Int (Lond). 2025 Dec 9;6(1):5. doi: 10.3892/mi.2025.289 (PMC12715461; doi:10.3892/mi.2025.289)
Supplement: Comparison of the performance AI vs. that of human experts across different specialties. [file Supplementary_Data.pdf]

Table SI. Comparison of the performance AI vs. that of human experts across different specialties.

Specialty	Specific task/study	AI performance	Human expert performance	Sample size	(Refs.)[a]
Cardiology (ECG)	Detecting structural heart disease from 12-lead ECG (EchoNext)	Accuracy, 77.3%; sensitivity, 72.6%; specificity, 80.7%	Mean cardiologists' accuracy, 64.0%; sensitivity, 61.1%; specificity, 66.1%	150 ECGs	(5)
Radiology (chest X-ray)	Atelectasis detection (CNN on NIH dataset)	AUC, 0.862 (95% CI, 0.825-0.895)	Radiologists' AUC, 0.808 (95% CI, 0.777-0.838)	112,120 chest x-rays	(32)
Oncology imaging/breast cancer	Breast cancer detection (neural network)	Sensitivity, 0.85	Physicians sensitivity 0.77	120,950 patients	(33)
Pathology (prostate biopsies)	Detect neoplastic lesions (Paige/Galant)	ROC AUC, ~0.99; sensitivity, 97.7%; specificity, 99.3%	Outperformed experts in narrow tasks	1,876 prostate biopsies	(14)
Dermatology	Skin lesion classification (CNN trained on dermoscopic images)	Mean sensitivity, 87.5%; mean specificity, 60%	Outperformed many dermatologists in head-to-head	12,378 dermoscopic images; compared vs. dermatologists (136/157 outperformed)	(26)
Ophthalmology (AMD classification)	DeepSeeNet staging of AMD	Accuracy, 0.671; sensitivity, 0.590; specificity, 0.930	Retinal specialists: Accuracy, 0.599; sensitivity, 0.512; specificity, 0.916	Trained on 60,000 images; tested on 900 images	(30)
Echocardiography	Automated LVEF assessment (non-inferiority trial)	Substantial assessment error, 16.8% (AI group)	Substantial assessment error, 27.2% (sonographers)	3,769 echocardiographic studies (AI, n=292/1,740; sonographer, n=478/1,755)	(95)

[a]The references cited in the table can be found in the reference list in the main manuscript. AI, artificial intelligence; AUC, area under the ROC curve; CI, confidence interval; CNN, convolutional neural network; ECG, electrocardiogram; ROC, receiver-operating characteristic; AMD, age-related macular degeneration; LVEF, left ventricular ejection fraction.

Table SII. Key clinical applications of AI by specialty.

Specialty	Key clinical applications	Example outcomes	Sample size	(Refs.)[a]
Radiology/Imaging	Lesion detection, segmentation, triage/automation, radiomics (fMRI, PET/CT)	Improved diagnostic accuracy, triage; some tools high specificity	Varies by study	(6-8,10-12)
Pathology/digital pathology	Pre-screening slides, tumor detection, Gleason grading, prognostic models	High ROC AUC for some tasks; workflow gains	1,876 prostate biopsies; challenge with >10,600 biopsies; MMAI trained on 16,204 slides (5,654 patients)	(13-15)
Dermatology	Dermoscopic image classification, triage/referral decision support	AI matched/exceeded dermatologists in some datasets	2,201 images in one study (240 used in human trials); CNN trained on 12,378 images (head-to-head with 157 dermatologists)	(19,21-27)
Ophthalmology	Diabetic retinopathy screening (IDx-DR, EyeArt), OCT + fundus analysis	High sensitivity/specificity for screening tools	IDx-DR: Tested on 900 patients; EyeArt: ~850,908 images from 101,710 patients; DeepSeeNet: trained on 60,000 images, tested on 900 images	(29-31)
Cardiology	ECG interpretation, echocardiography automation, AI-QCT, RPM, MACE prediction, AI-QCA	Improved detection of LVH; automated quantification (EchoGo Core); FDA-cleared tools	Examples: AI-QCT CREDENCE cohort, 303 patients; QCG cohort, 1,254 patients with acute HF; pooled study, 66,479 participants for LVH analysis; 400 patients in FLASH trial	(93-97,100,102,107-110)
Surgery/robotics	Pre-op planning, intra-op guidance, semantic segmentation, risk prediction	Reduced blood loss; AR guidance under investigation	Not specified	(61-66,69-76)
Decision support and precision treatment	Clinical decision support, TDM, individualized dosing (warfarin, CURATE AI)	AI improved individualized dosing and predictions	Warfarin dosing studies and CURATE AI examples reported (sample sizes varied)	(36-39,54,55)

Public health/surveillance	Outbreak detection, population surveillance	Early detection systems	Varies by system	(48,121)
Workflow	EHR NLP, scheduling, virtual assistants, ambient scribe	Reduced admin workload; improved documentation efficiency	Sample sizes vary	(118,119,188,189)

[a]The references cited in the table can be found in the reference list in the main manuscript. AI, artificial intelligence; fMRI, functional magnetic resonance imaging; PET/CT, positron emission tomography/computed tomography; AUC, area under the ROC curve; ROC, receiver-operating characteristic; MMAI, multimodal artificial intelligence; CNN, convolutional neural network; OCT, optical coherence tomography; FDA, Food and Drug Administration; LVH, left ventricular hypertrophy; AI-QCT, AI-enabled quantitative coronary computed tomographic angiography; RPM, remote patient monitoring; MACE, major adverse cardiac events; AI-QCA, AI-based quantitative coronary angiography; HF, hear failure; AR, augmented reality; TDM, therapeutic drug monitoring; EHR, electronic health record; NLP, natural language processing; DR, diabetic retinopathy.

Table SIII. Regulatory frameworks and guidelines for AI in healthcare by region.

**Region/regulator**	**Key frameworks/guidance**	**Insights**	**(Refs.)[a]**
USA (FDA)	FDA AI/ML device framework; lifecycle strategy	Emphasis on post-market monitoring, iterative performance monitoring	(147,185)
European Union/EMA	MDR; EU AI Act	Supervision obligations for high-risk AI; gaps noted (e.g., DermAssist self-certified Class I)	(149,157,173,174)
UK	NHS guidelines	Concerns over approval without robust validation	(138,139)
Global/WHO	WHO guidance on transparency and accountability	Calls for inclusive standards and external testing	(144,153)
Data protection regimes	GDPR, HIPAA	Privacy, consent, data ownership concerns	(137,144,158)
Clinical evaluation	DECIDE-AI; shadow deployment recommendations	Recommend phased clinical testing and ongoing audits	(144,147)

[a]The references cited in the table can be found in the reference list in the main manuscript. AI, artificial intelligence; FDA, Food and Drug Administration; EMA, European Medicines Agency; MDR, Medical Device Regulation; EU AI Act, European Union Artificial Intelligence Act; WHO, World Health Organization; GDPR, General Data Protection Regulation; HIPAA, Health Insurance Portability and Accountability Act; DECIDE-AI, phased clinical evaluation guidance for AI.

Table SIV: Future AI technologies and expected timeline for clinical implementation.

**Technology/area**	**Timeline/status**	**Key notes**	**(Refs.)**[a]
AI in imaging and screening (Transpara, INSIGHT MMG, ProFound AI)	Prospective trials	Encouraging prospective trial results; moving to deployment	(162-165)
Workflow optimization/triage systems	Near-term adoption	Risk-based triage to prioritize cases	(166)
Multi-omics integration (genomics + proteomics + imaging)	Early stages; requires harmonization	Standardization and federated learning needed	(167-171)
Mixed reality/AR/VR intraoperative guidance	Research/development	AI + AR/VR could enable intraoperative guidance	(61,177)
Semi-autonomous/autonomous robotic surgical systems	Early research/early clinical translation	Human-in-the-loop remains standard	(69,87,82)
Digital twins/real-time image guidance	Research/not yet widely available	Dependent on high-fidelity data; emerging	(83,179,181)
3D bioprinting and personalized organ manufacture	Future research	Research stage	(66)
Micro- and nanorobots for drug delivery	Future concept	Longer-term innovation	(66)
Education, regulation and reimbursement changes	Ensuing 5-10 years	Training and regulatory shifts expected	(183-187)

[a]The references cited in the table can be found in the reference list in the main manuscript. AI, artificial intelligence; AR/VR, augmented reality / virtual reality; MMG, mammography; multi-omics, integration of genomics/proteomics/imaging; 3D, three-dimensional.

Table SV. AI tools and systems in contemporary medicine

Tool	Domain	Notes and metrics	(Refs.)[a]
EchoNext (deep-learning ECG)	Cardiology: detect structural heart disease from 12-lead ECG	Outperformed cardiologists (accuracy 77.3 vs. 64.0%) Sample size - 150 ECGs read by 13 cardiologists	(5)
IDx-DR	Ophthalmology: Autonomous diabetic retinopathy screening	Sensitivity, ~87.4%; specificity, ~89.5%; FDA-approved Tested on 900 patients across 10 primary care settings	(30,31)
EyeArt 2.0	Ophthalmology: Fundus DR screening	91.3% sensitivity/91.1% specificity Sample size ~850,908 images from 101,710 patients	(30)
DeepSeeNet	Ophthalmology: AMD staging from color fundus images	Outperformed retinal specialists on test set Trained on 60,000 images; tested on 900	(30)
Paige prostate	Pathology: Prostate cancer pre-screening	FDA-authorized (2021); 97.7% sensitivity/99.3% specificity Sample size-1876 prostate biopsies	(14)
Galant prostate (IBEX)	Pathology: Prostate detection	97.7% sensitivity/99.3% specificity reported Sample size: 1,876 prostate biopsies	(14)
Transpara/INSIGHT MMG/ProFound AI	Radiology: Mammography screening support	Prospective trials Sample sizes vary by trial	(162-164)
AI-QCT (Cleerly Inc.)	Cardiac CT: Plaque quantification	Automated coronary plaque quantification CREDENCE cohort: 303 patients	(96)
HeartFlow FFRct Analysis	Cardiology: Coronary physiology modeling	FDA-cleared for CAD diagnosis	(110,112)
Viz.ai (Viz RV/LV)	Imaging triage - RV/LV ratio, PE triage	FDA-approved triage platform (K221100)	(109,110)
Aidoc BriefCase-Triage	Imaging triage (aortic dissection prioritization)	Facilitates rapid prioritization (K251406)	(110,111)
Ultromics EchoGo Core	Echocardiography: Automated cardiac measures	Cleared under FDA 510(k) K191171; produces EF and volumes	(107,108)
Cydar EV Maps	Intraoperative alignment/navigation (aneurysm repair)	Aligns preop imaging with real-time fluoroscopy (FDA-approved)	(75)
CURATE AI	Precision oncology: Individualized chemo dosing	Adjusted chemo doses to improve response and reduce toxicity	(55)
Warfarin dosing algorithms (RNN)	Pharmacotherapy: Individualized dosing	Algorithms outperformed clinicians	(90,103)
Caption guidance	Point-of-care ultrasound assistance	FDA-approved system guiding non-specialists	(92,105)

[a]The references cited in the table can be found in the reference list in the main manuscript. AI, artificial intelligence; ECG, electrocardiogram; IDx-DR, autonomous diabetic retinopathy screening system; FDA, Food and Drug Administration; DR, diabetic retinopathy; AMD, age-related macular degeneration; AI-QCT, AI-enabled quantitative coronary computed tomographic angiography; CAD, coronary artery disease; RV/LV, right ventricle/left ventricle; EF, ejection fraction; FFRct, fractional flow reserve from CT; RNN, recurrent neural network.
